# Recipients of 2021 CSSA Editor's Citation for Excellence Named

**DOI:** 10.1002/tpg2.20231

**Published:** 2022-05-24

*The editorial board of The Plant Genome is pleased to announce the recipients of the 2021 Editor's Citation for Excellence. These awards recognize the outstanding professional commitment and dedication of volunteer reviewers and/or editors who, through their excellent insights and comments, have helped maintain the high standard and quality of papers published in the journal. Recipients were nominated based on their thorough, competent, and timely reviews or editing of manuscripts. Each will receive a certificate of appreciation, an ASA‐CSSA‐SSSA gift certificate, and recognition in CSA News*.

## Associate Editors

Rajeev Varshney, Murdoch University, Australia

Nils Stein, Leibniz Inst. of Plant Genetic and Crop Plant Research IPK, Germany

## Reviewer

Shuyu Liu, Texas A&M AgriLife Research, USA

